# Innovative fast and low-cost method for the detection of living bacteria based on trajectory

**DOI:** 10.1038/s41598-025-95069-9

**Published:** 2025-05-13

**Authors:** Paul Perronno, Julie Claudinon, Carmen Senin, Serap Elçin-Guinot, Lena Wolter, Olga N. Makshakova, Norbert Dumas, Dimitri Klockenbring, Joseph Lam-Weil, Vincent Noblet, Siegfried Steltenkamp, Winfried Römer, Morgan Madec

**Affiliations:** 1 ICube Laboratory, UMR 7357 (CNRS/University of Strasbourg), 67400 Illkirch-Graffenstaden, France; 2 Faculty of Biology, University of Freiburg, 79104 Freiburg, Germany; 3 Signalling Research Centres BIOSS and CIBSS, University of Freiburg, 79104 Freiburg, Germany; 4 OPHARDT Hygiene-Technik GmbH, 47661 Issum, Germany; 5 Spemann Graduate School of Biology and Medicine, University of Freiburg, 79104 Freiburg, Germany

**Keywords:** Bacteria identification, Optical detection, Trajectory analysis, Machine learning, Pathogen detection, Bacterial motility, Optical imaging, Lab-on-a-chip, Software, Information technology

## Abstract

Detection of pathogens is a major concern in many fields like medicine, pharmaceuticals, or agri-food. Most conventional detection methods require skilled staff and specific laboratory equipment for sample collection and analysis or are specific to a given pathogen. Thus, they cannot be easily integrated into a portable device. In addition, the time-to-response, including the sample collection, possible transport to the measurement equipment, and analysis, is often quite long, making real-time screening of a large number of samples impossible. This paper presents a new approach that better fulfills industry needs in terms of integrated real-time wide screening of a large number of samples. It combines optical imaging, object detection and tracking, and machine-learning-based classification. Three of the most common bacteria are selected for this study. For all of them, living bacteria are distinguished from inert and inorganic objects (1 μm latex beads) based on their trajectory, with a high degree of confidence. Discrimination between living and dead bacteria of the same species is also achieved. Finally, the method successfully detects abnormal concentrations of a given bacterium compared to a standard baseline solution. Although there is still room for improvement, these results provide a proof of concept for this technology, which has strong application potential in infection spread prevention.

## Introduction

The detection of pathogens is a major concern in many lines of business such as biology^1^, agri-food^2^, environment monitoring^3^ or medicine^4–6^. Regarding this last field, infections occurring during a hospital stay, called nosocomial infections, are nowadays a significant challenge in healthcare. In Germany it was estimated that 400,000 people per year were suffering from nosocomial infections (considering the data from^4,5^) in 2022. In France, it represents 750,000 infections per year^6^.

Various infection identification techniques exist, but they are usually expensive, time-consuming, and/or labor-intensive. Cultivation of these pathogens in a laboratory environment is a widely used method for bacterial or fungal identification^7,8^, However, this technique is time-consuming: the incubation step alone can take up to 7 days until visible growth can be observed^7^, and that usually underestimates reality^9,10^.

Today, the Polymerase Chain Reaction (PCR) is the gold standard for infection detection^11^. It is a repetitive amplification process of the deoxyribonucleic acid (DNA) of a specific pathogen in the sample. The popularity of this technique increased with the appearance of Quantitative PCR (Q-PCR), enabling quantification of the targeted pathogen^12,13^. However, PCR is very specific to the target DNA of a given microorganism.

Other infection detection techniques include immunological assays. These tests aim to detect a specific molecule through a labeled capturing agent specific to one target^14^. These methods have a low time-to-response span but are usually less sensitive than PCR. Alternative techniques can even be used to confirm the results^15^. Despite the advantages, serological assays are very specific and are hard to apply to unknown samples.

In recent years, miniaturized electrochemical sensors emerged. These sensors enable high-throughput and real-time analysis with reduced sample volumes. They often require a specific functionalized surface or affinity capture^15^.

A label-free bacterial detection technique was recently developed. It is based on bright-field microscopy images, simple image processing, and classification algorithms. The classification relies on the morphology of the objects detected^16^. However, making this technology portable requires the transition from a laboratory microscope (e.g. EVOS FL inverted digital fluorescence microscope from Advanced Microscopy Group (AMG) used in^16^) to an integrable and low-cost microscope, which is very challenging, in particular, because of the low resolution of the images which, in turn, reduces the accuracy of the estimation of morphological parameters, i.e. the features of the machine-learning algorithm. As optical detection remains a promising method for producing low-cost, easily integrated systems, we have been investigating approaches that release constraints on image quality and resolution. One of them is the identification of bacteria using their trajectories as a discriminant factor.

In a liquid environment, living bacteria have different swimming behaviors. Their behavior depends among others on the shape of the bacterium, the presence or absence of flagella, their number, and their positions^17^. Apart from auto-generated movements, bacteria can undergo passive displacement caused by external forces, such as Brownian motion^18^.

Recently, the first tool using trajectories of particles to differentiate objects was developed^19^. The measurement of Brownian motion on tracked particles was used to separate between extracellular vesicles and bacteria. However, this tool could not differentiate particles of the same type such as two species of bacteria. The Brownian motion was only used to calculate the size of the particles and sort them based on this value. Trajectory detection is widely spread across many fields. Many objects can be tracked, people^20^, cars^21^, or particles^22^. Applications are wide such as predicting behaviors^21^, better-understanding sports phenomena^23^, or sorting objects^24^, and usually, the algorithms are quite specific to the application.

This study serves as a proof of concept for the detection and identification of living bacteria in Phosphate Buffered Saline (PBS) based on their trajectories using movies acquired with a low-cost microscope. The processing pipeline includes the identification of trajectories from a movie, the calculation of 11 features of these trajectories, and the application of a supervised machine-learning algorithm (Random Forest) to discriminate living bacteria from dead ones and other inert and inorganic objects (bacteria-sized beads), referred to simply as inert objects in the following. The study focuses on 3 species of bacteria often involved in infections, namely *Escherichia coli* (*E. coli*), *Pseudomonas aeruginosa* (*P. aeruginosa*), and *Staphylococcus aureus* (*S. aureus*)^25^. Additionally, 1 μm latex beads were used as a model for other inert objects. Acquisitions were made in a microfluidic chamber filled with the sample under analysis.

## Results

The goal of this study is to identify particles based on their trajectories. To this end, an experimental set-up and a complete data processing pipeline were designed. The experimental set-up is composed of a small custom-made microscope made of a 40x Zeiss objective, a lens, a green LED as a light source, and a Ximea camera. Several optical components were tested beforehand to design this microscope. The hardware configuration we chose for the experiment presented in this paper is the one we believe to be the best tradeoff between image quality, resolution, and price. All samples were imaged using the software supplied by Ximea in a straight-channel microfluidic chip µ-Slide I from Ibidi. This study focuses on the individual movements of particles. Measures, like sealing the chips, were taken to reduce external movements and flow as much as possible. However, despite these precautions, a residual flow still exists. If necessary, the residual flow was removed through software corrections. Details and references are provided in the Methods section.

**Fig. 1:**
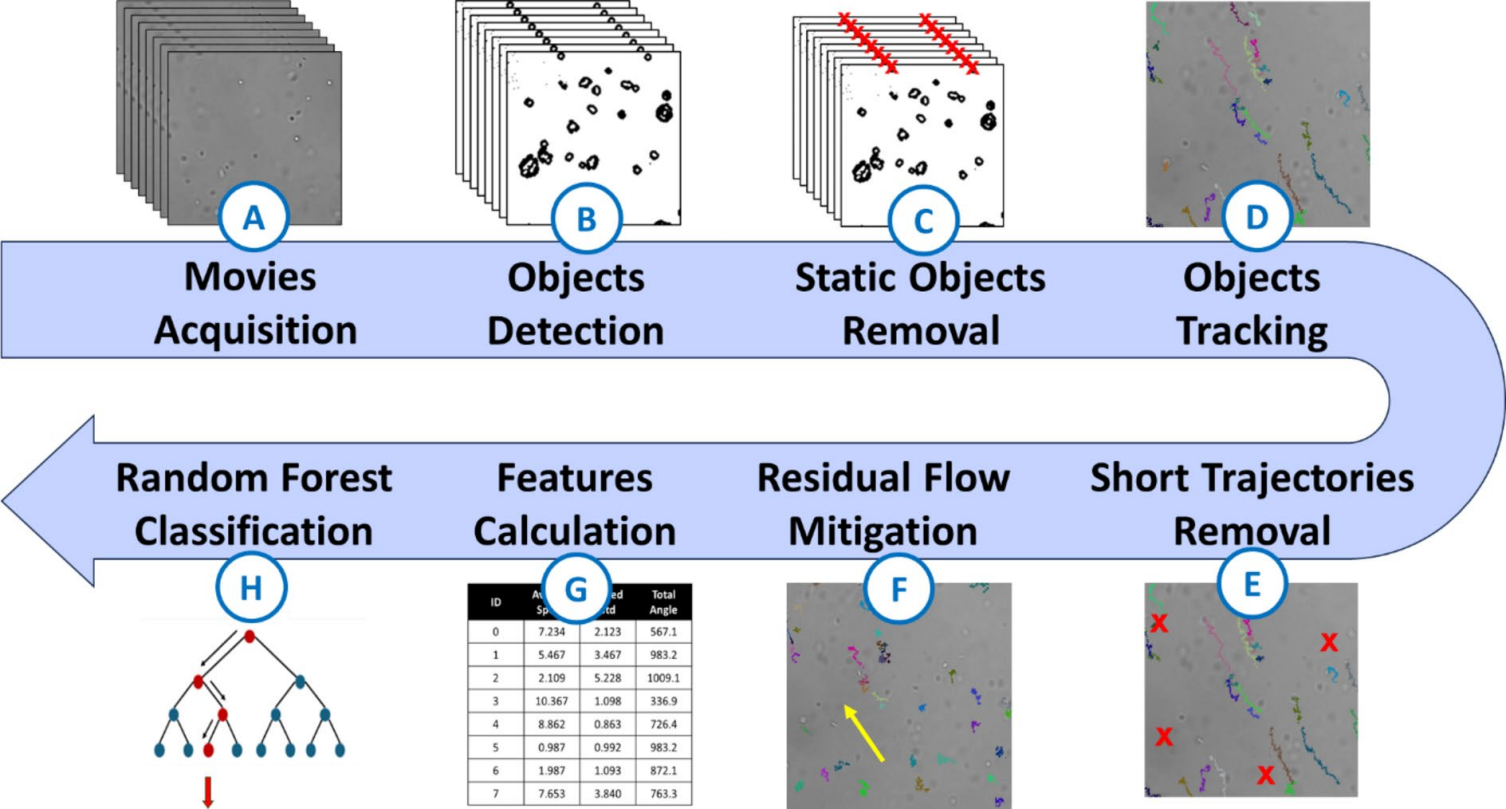
The architecture of the 8-stage analysis pipeline. First, a 30-second video of the sample is acquired (A). Then, all particles are detected in every frame of the movie (B). Next, non-moving contaminating particles that appear in each frame but do not belong to the sample are removed (C). A particle tracking algorithm is then used to trace back the trajectories of the different objects in the video (D). Small trajectories, which are not representative, are then removed (E). Detected objects might also have undergone a residual flow in the chamber (i.e., resulting in the movement of the medium) which affects all trajectories and could hide individual motion. The next step is to estimate and subtract this residual flow from all trajectories (F). Eleven features are then calculated for each trajectory (G). These features are given as inputs to the classifier which predicts a label for each trajectory (H). For each step, further details on the algorithm are described in the Methods section.

**Fig. 2:**
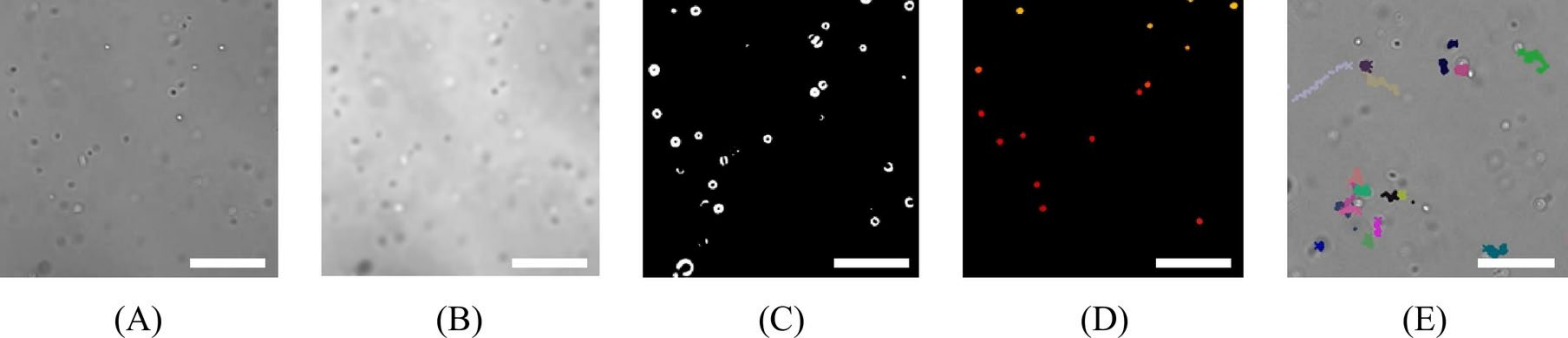
Results of image processing for acquisitions of *E. coli* made in PBS. (**A**) Raw image of living *E. coli* in PBS suspension. (**B**) The same image after processed by a Gaussian filter and levels adjustment. Black and white dots indicate particles in the sample, here *E. coli*. (**C**) Binary masks used to identify and label objects in (B). White dots indicate *E. coli* which are above the detection threshold, black is the background. (**D**) Labeled image (pseudo-color) after segmentation. Colors are arbitrarily chosen and indicate what the algorithm identified as objects in (**C**). Each object has a different color. (**E**) Image of *E. coli* after the particle tracking algorithm. Colors are arbitrarily chosen and indicate the different trajectories of the objects detected in (**D**). Size of images: 540 × 540 pixels (cropped from the 2464 × 2056 full-scale images). Scale bar: 20 μm.

**Fig. 3:**
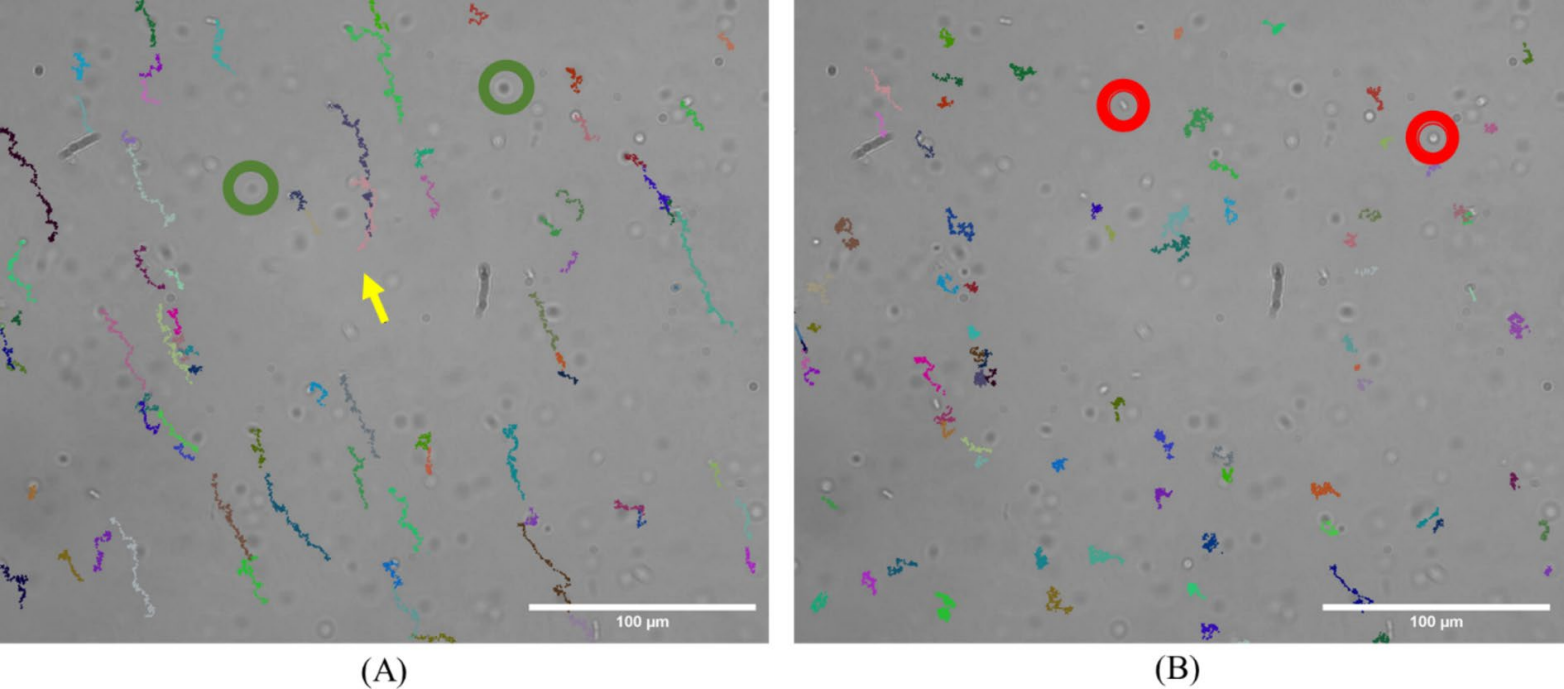
Illustration of the residual flow mitigation method. A video was captured of a solution containing only *P. aeruginosa* at a concentration of approximately 10^5^ particles/mL. Images were acquired using a custom-built microscope (40x objective). The figure corresponds to the last frame of the video, with the trajectories of each detected particle in the video. Each trajectory is represented in an arbitrary color. Particles outside the focal plane were not tracked, and therefore, no lines represent their trajectories. Red and green circles indicate particles outside the focal plane of the imaging system, with red circles representing particles above the focal plane and green below it. The yellow arrow in (**A**) indicates the average trajectory, which is subtracted from all trajectories of (**A**) to produce the corrected trajectories of (**B**). (**A**) shows trajectories before the average flow undergone by all particles was removed. (**B**) shows the trajectories of the same particles after the average flow has been removed. Since the trajectory colors were arbitrarily assigned based on the order of detection, a same particle has different color in (**A**) and in (**B**). Scale bars: 100 μm.

Acquired movies then went through the data processing pipeline, as shown in Fig. 1. The pipeline includes the detection of objects in each image, the construction of trajectories from these frame-by-frame objects, the calculation of the features of these trajectories, and the classification. Figure 2 depicts some intermediate results of the pipeline. Eleven features are taken into consideration for the classification process. The features were selected based on literature research on the various trajectories of microorganisms^17,26–29^ and their most discriminating factors (e.g. average speed, linearity of the trajectory, average rotation angle). Corrections are applied at various stages of the pipeline to remove artifacts (treated as background) and correct the trajectories by calculating and subtracting the residual flow. The results of this residual flow mitigation are presented in Fig. 3. Details about all algorithms used for the different stages of the pipeline are given in the Methods section.

**Fig. 4:**
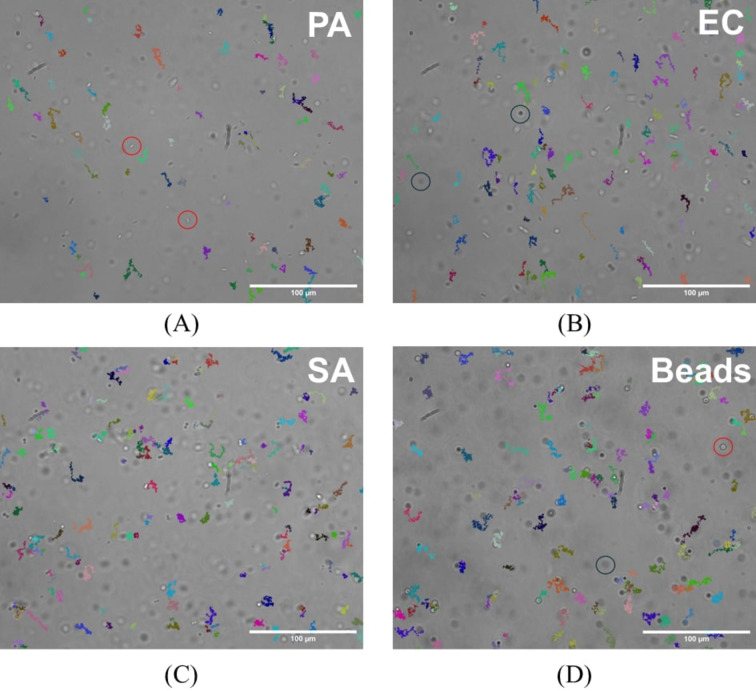
Trajectories detected for the three bacteria studied and for beads. Images were acquired with a homemade microscope (40x objective). The concentration of particles is around 10^5^ particles/mL for all images. Particles circled in red and black are objects contained in the sample. Objects circled in red are above the focal plane of the imaging system and particles circled in black are below the focal plane. Particles outside the focal plane are not tracked. Each colored line indicates a trajectory detected from the movie where the images come from. Residual flow mitigation has been applied to displayed trajectories. (**A**) shows trajectories detected for *P. aeruginosa*, (**B**) for *E. coli*, (**C**) for *S. aureus*, and (**D**) highlights trajectories of beads. Colors are arbitrarily chosen. Scale bars: 100 μm.

Trajectories detected for each bacterial species and beads for an equivalent period are depicted in Fig. 4. The presented trajectories are from randomly chosen movies. Examples of trajectories for objects studied, namely living *P. aeruginosa*, living *E. coli*, living *S. aureus*, and beads, are shown in Fig. 4A, B, C, and D respectively. The trajectories displayed are trajectories after the residual flow mitigation.

### Classification of living Bacteria versus beads

First, we assessed the possibility of differentiating living bacteria from inert objects of similar size. To this end, each bacterium was tested against 1 μm latex beads, which were used as models for inert objects. As for most of the experiments described below, four samples were prepared for each type of bacteria: one that contains only objects of one type to identify (here living bacteria), one that contains only objects of the other type (here beads), and two that contain a mixture of both objects with a ratio of 70:30 and 30:70, respectively. The two first samples were used for the training and the verification of the machine-learning algorithm while the latter two were used to assess its performance. For each type of bacteria, a classifier was trained to distinguish between two classes: the living bacteria and beads.

After the random forest was trained, it is possible to access the feature importance of the model, i.e. the frequency at which a given feature is used in decision trees. For all experiments, the features used most frequently by classifiers to make decisions are the ones related to the speed: the average speed (45% ± 5%), the standard deviation of the speed (25% ± 3%), and the speed slope (12% ± 4%).

**Fig. 5:**
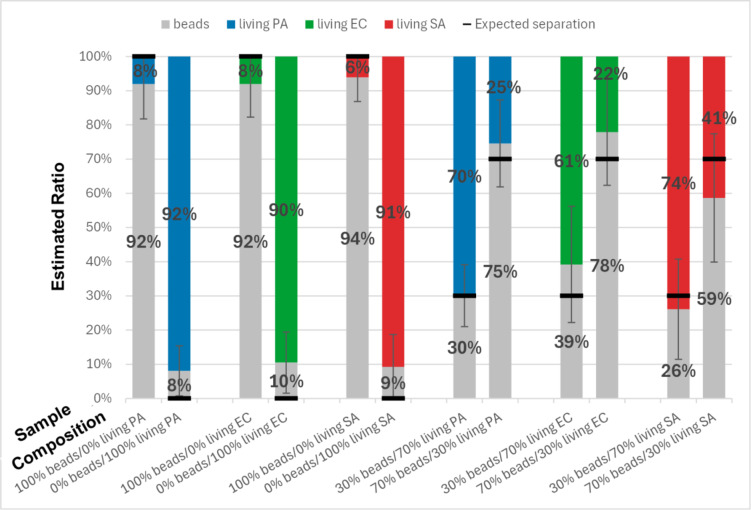
Histogram representation of the results of the classifiers applied on living bacteria and beads. Each bar represents the classification results for a different actual ratio displayed at the bottom of each column. Each ratio has been tested with a specific binary classifier trained to differentiate between beads and the corresponding living bacteria. Each class, i.e. type of bacteria or beads, is represented by a specific color. Beads are shown in grey, *P. aeruginosa* in blue, *E. coli* in green, and *S. aureus* in red. The black line on each bar indicates the expected separation between the two classes, i.e. the actual ratio. The values featured here are the average results obtained over 30 videos per ratio (180 videos in total). These videos were acquired over 3 biological replicates (10 videos per replicate). The whiskers indicate the standard deviation obtained over the results of the 30 videos of the tested ratio. From left to right, the standard deviations vary from 7–19%, depending on the experiment. In this graph, PA stands for *P. aeruginosa*, EC for *E. coli*, and SA for *S. aureus*.

The classification of all 3 living bacteria studied versus the beads is shown in Fig. 5. In this figure, as for all figures hereafter, each bar corresponds to an experiment and gives the expected ratio (according to the sample preparation) and the ratio estimated by the algorithm for each class. For instance, the first bar on the left shows that the sample is expected to contain 100% of beads but the algorithm estimates it contains 8% of *P. aeruginosa* and 92% of beads. The results presented in all the following figures were obtained on videos different from those used to train the classifiers.

The left half of Fig. 5 demonstrates the effectiveness of training. Estimations obtained by the algorithm are close to the expected ratios for all investigated samples. The highest learning error is observed for living *E. coli* (10%). The right half of Fig. 5 shows that the trained algorithm can predict effectively and quantitatively the ratio of bacteria/beads in the sample. In the worst case (last column), a difference of 11% between the actual and the estimated ratio is observed. This value can be explained both by the misclassification error described hereabove, as well as errors in mixture preparation, estimated at 8% (the way this value is estimated is explained in the Methods section).

From these results, we can conclude that each tested classifier can differentiate between a specific bacterium and inert objects for 3 bacterial species that have different motion behaviors. *E. coli* and *P. aeruginosa* are rod-shaped bacteria with at least one flagellum surrounding their membrane^17,27,28^. Moreover, *P. aeruginosa* is known to be more active than *E. coli*^27,28^. In contrast, *S. aureus* has a morphology very similar to the beads: it is a spherical particle with no flagella to induce motion^26^. Consequently, the detection of *S. aureus* versus beads is expected to be less efficient than the detection of the other bacteria. Nevertheless, the misclassification rate for *S. aureus* is not worse than the ones for *E. coli* and *P. aeruginosa*.

### Classification of dead bacteria versus beads

The second set of experiments aimed at determining if this technique could also be applied to dead bacteria. At first glance, dead bacteria were expected to behave like inert objects, but it can assumed that their morphology (shape, presence of flagella), which is not affected by the killing technique used in this study (see protocol details in the Methods section), might give them trajectories that differ from beads. To check this hypothesis, twelve new samples were prepared with the same composition as described above, except that dead bacteria replaced living ones. The samples were tested with three new classifiers trained on each type of bacteria.

**Fig. 6:**
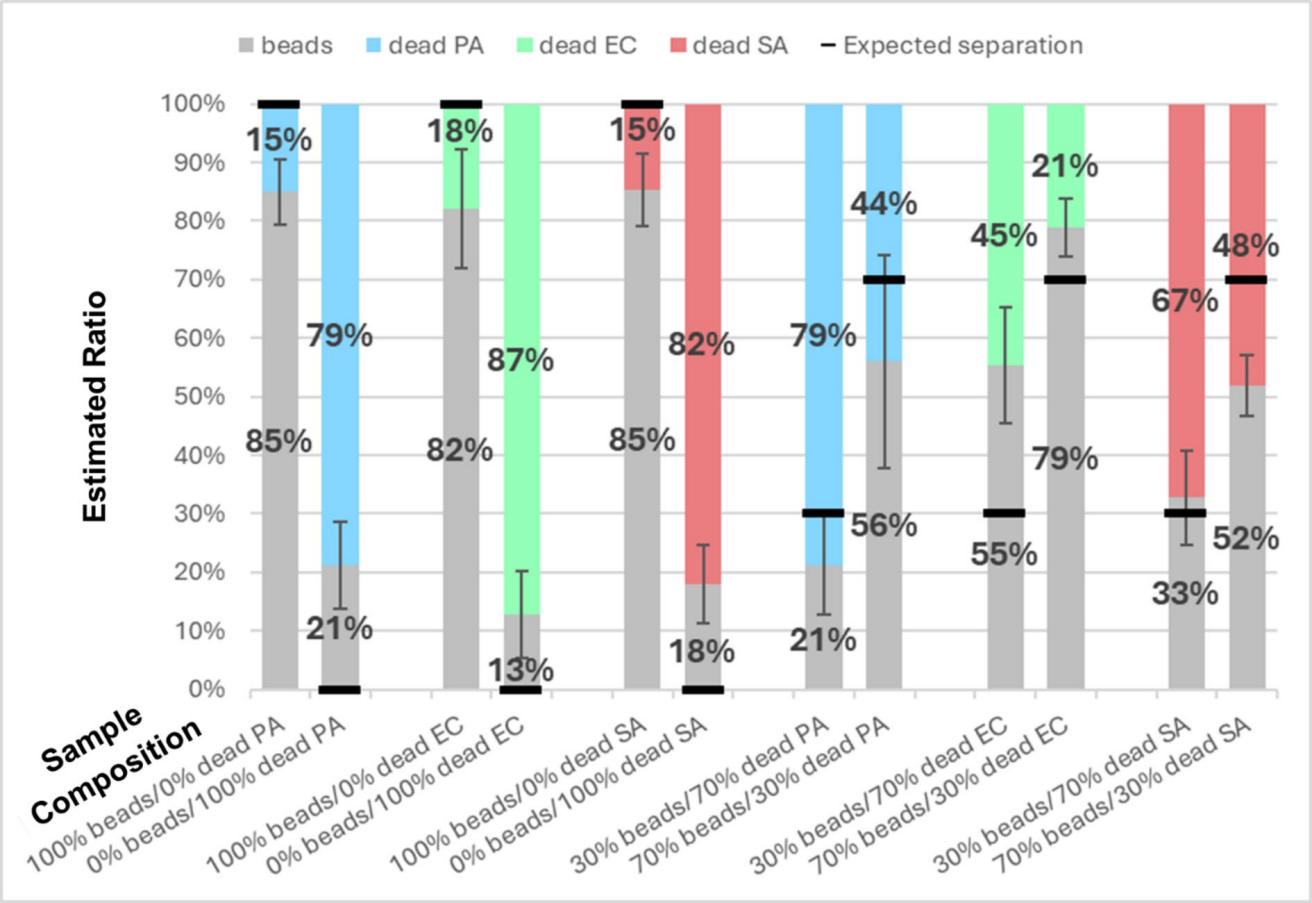
Histogram representation of the results of the classifiers applied to dead bacteria and beads. Each bar represents the classification results for a different ratio between beads on the one hand, and the studied dead bacteria on the other hand. The expected ratio for the given experiment is displayed at the bottom of each column, on the x-axis. Each type of particle (or class) is linked to a specific color. Beads are shown in grey, dead *P. aeruginosa* in pastel blue, dead *E. coli* in pastel green, and dead *S. aureus* in pastel red. The black line on each bar indicates the expected separation between the two classes based on the real ratio. The values featured here are the average results obtained over 30 videos per ratio (180 videos in total). These videos were acquired over 3 biological replicates (10 videos per replicate). The whiskers indicate the standard deviation obtained over the results from the 30 videos of the considered experiment. From left to right, the standard deviations vary between 6% and 18%, depending on the experiment. In this graph, PA stands for *P. aeruginosa*, EC for *E. coli*, and SA for *S. aureus*.

The classification results are presented in Fig. 6. It can be pointed out that the classification is less efficient for dead bacteria compared to living ones: the misclassification rate for pure samples increases from 9.5 to 17.3%. This is likely to be because dead bacteria move less than living ones, which increases the difficulty of distinguishing between them and beads based only on motion. Despite this inaccuracy, the expected trend is observed when comparing the four samples of the same bacterium: the estimated ratio between beads and dead bacteria increases when the actual ratio increases with gaps between the expected and the actual ratio ranging from 3 to 25% in the worst case (13% on average over the six mixtures). We conclude that the algorithm is also able to discriminate between dead bacteria and inert objects. Features importance analysis gives about the same tendency as for living bacteria: the three most frequently used features are the average speed (42% ± 7%), the standard deviation of the speed (21% ± 6%), and the speed slope (12% ± 7%).

### Classification of living bacteria versus dead bacteria

**Fig. 7:**
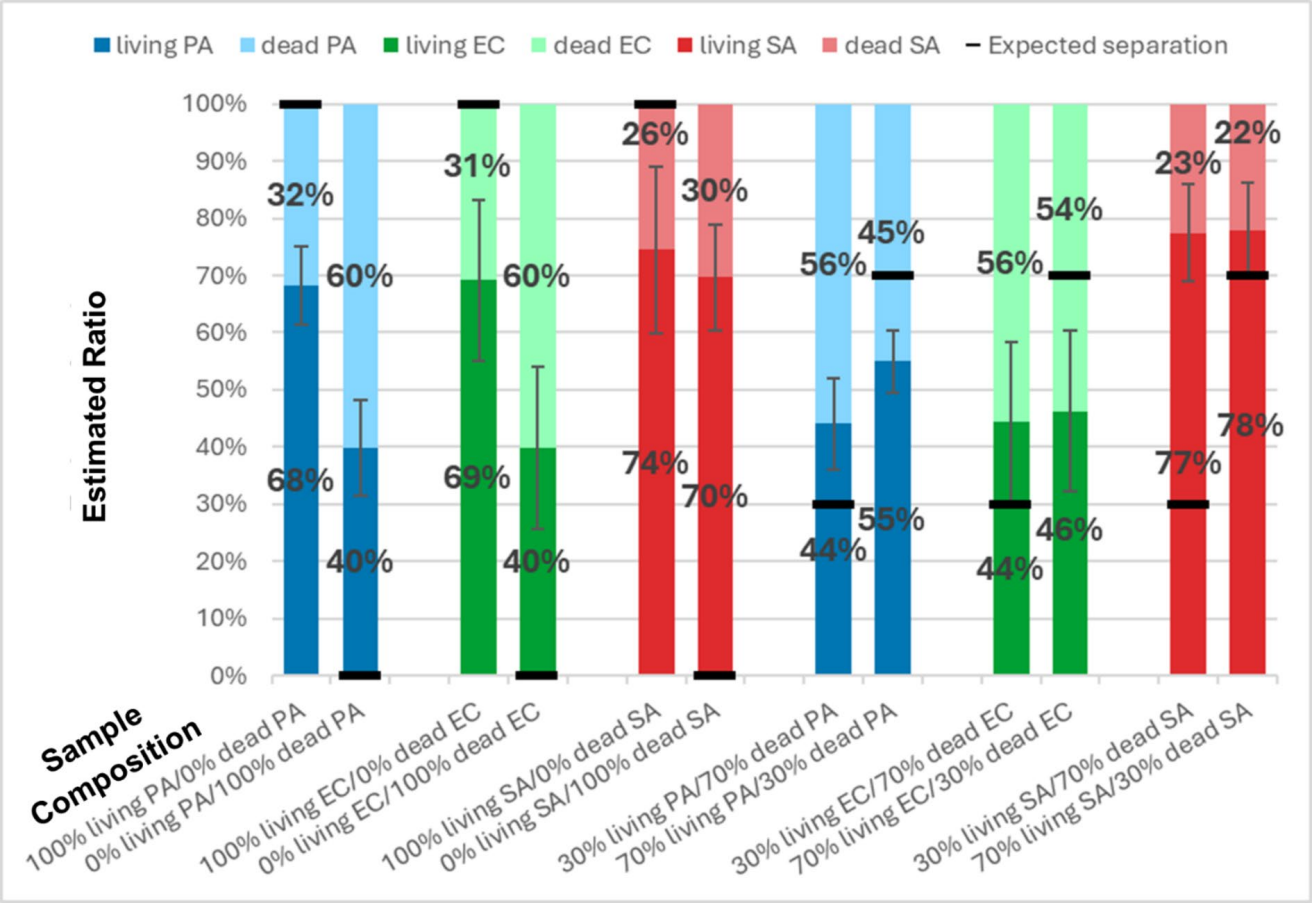
Histogram representation of the results of the classifiers applied to living bacteria and dead bacteria in separate samples. Each bar represents the classification results for a different ratio. The expected ratio for the given experiment is displayed at the bottom of each column, on the x-axis. Each type of particle (or class) is linked to a specific color. Living *P. aeruginosa* is shown in blue, dead *P. aeruginosa* in pastel blue, living *E. coli* in green, dead *E. coli* in pastel green, living *S. aureus* in red, and dead *S. aureus* in pastel red. The black line on each bar indicates the expected separation between the two classes based on the real ratio. The values featured here are the average results obtained over 30 videos per ratio (180 videos in total). These videos were acquired over 3 biological replicates (10 videos per replicate). The whiskers indicate the standard deviation obtained over the results from the 30 videos of the tested ratio. From left to right, the standard deviations vary between 7 and 14%, depending on the experiment. In this graph, PA stands for *P. aeruginosa*, EC for *E. coli*, and SA for *S. aureus*.

The third set of experiments aimed at comparing the trajectories of living and dead bacteria of the same microorganism. Twelve samples were prepared for this purpose, four for each bacterium: one sample that contains only living bacteria, one only dead bacteria, and two mixtures of dead and living bacteria with 70:30 and 30:70 ratios respectively. To sort the trajectories, 3 new binary classifiers were trained to compare one type of bacteria between its living and dead states.

Figure 7 presents the results obtained by the algorithm. The misclassification rate increased significantly, reaching up to 40% for *E. coli* and *P. aeruginosa*. However, the algorithm consistently identifies the majority of species in each sample as the true major species. Notably, *P. aeruginosa* exhibits a marked shift between its mixture samples whereas *E. coli* presents little to no change. As *P. aeruginosa* and *E. coli* have similar morphologies, the more active behavior of *P. aeruginosa* seems to explain these discrepancies.

The increase in misclassifications highlights the difficulty of differentiating between the motion behavior of a living bacterium from the behavior of the same, but dead bacterium. This increment means that the movements of dead and living bacteria have similarities. However, as explained before, *P. aeruginosa* is a bacterium prone to movements when alive, and dead bacteria should not be able to create movements on their own, since their metabolism has stopped. This suggests that dead bacteria might be able to keep a certain motion behavior, even without metabolic activities.

For *S. aureus*, the prediction is about the same whatever the ratio of living and dead bacteria in the sample. The classification is thus inefficient. Movements of *S. aureus* have been studied extensively. Although they do not have a flagellum, *S. aureus* can move only by gliding on the surfaces^29^. This finding was confirmed through confocal imaging observations (results not shown). However, in the context of this study, the focal plane is located in the middle of the microfluidic chip. Thus, the bacteria observed in the videos are therefore far from any surface and can thus be considered as non-motile. The trajectories of *S. aureus* are therefore mostly defined by their morphology, which is, for the killing techniques used in this study (see Methods section for more details), the same whether dead or alive. This explains the poor results obtained when trying to distinguish between dead and alive *S. aureus* (Fig. 7), at least in comparison with *E. coli* and *P. aeruginosa*, which, although they also maintain their morphology, lose their motility upon death.

Feature importance analysis for each living-vs-dead classifier reveals differences from one type of bacteria to the other. For *E. coli* and *P. aeruginosa*, the average speed remains the most frequently used feature, with 27% ± 6% and 17% ± 3% respectively. For *E. coli*, the standard deviation of the speed (17% ± 4%) and the speed slope (12% ± 2%) are still in the second and third place. For *P. aeruginosa*, the length of the trajectory comes in second place (13% ± 2%). All other features have a frequency below 10% with a significant variation from one set of training videos to the other and can thus not be ranked. For *S. aureus*, the ranking of feature importance is topped by the total angle (30% ± 3%), the length of the trajectory (21% ± 3%), and the average speed (13% ± 2%).

Two key observations emerged from these experiments. First, the most important features of the living-vs-dead classifiers differed from those used for the bacteria-vs-beads classifiers. Second, the disparity between the most and least frequently used features was smaller for the living-vs-dead classifiers. This observation confirms that the trajectories of living and dead bacteria are more similar than when compared to beads. This makes the classification more challenging and forces the classifiers to use other, and probably less relevant, features, which are reflected in the classification results.

### Application example: detection of a contamination

The ultimate goal of the project in which this work was carried out is to create a system capable of alerting a person about potential contamination during the hand sanitization process. This contamination is defined by a high quantity of a pathogenic bacterium out of the crowd on the hand. To assess the suitability of the proposed method for this application, we conducted a new experiment that included the analysis of a mixture (called standard mixture in the following) of uncharacterized bacteria (in various proportions) to create a baseline. To closely mimic real-world conditions, the standard mixture is composed of microorganisms obtained by the cultivation of a hand bacterial population. Then, a sample composed of this standard mixture and a sample containing only *P. aeruginosa* were prepared. A new classifier was trained with videos acquired from these two samples. Then, a third sample composed of *P. aeruginosa* and the standard mixture at 80% and 20% respectively, was also prepared and processed by the classifier.

**Fig. 8:**
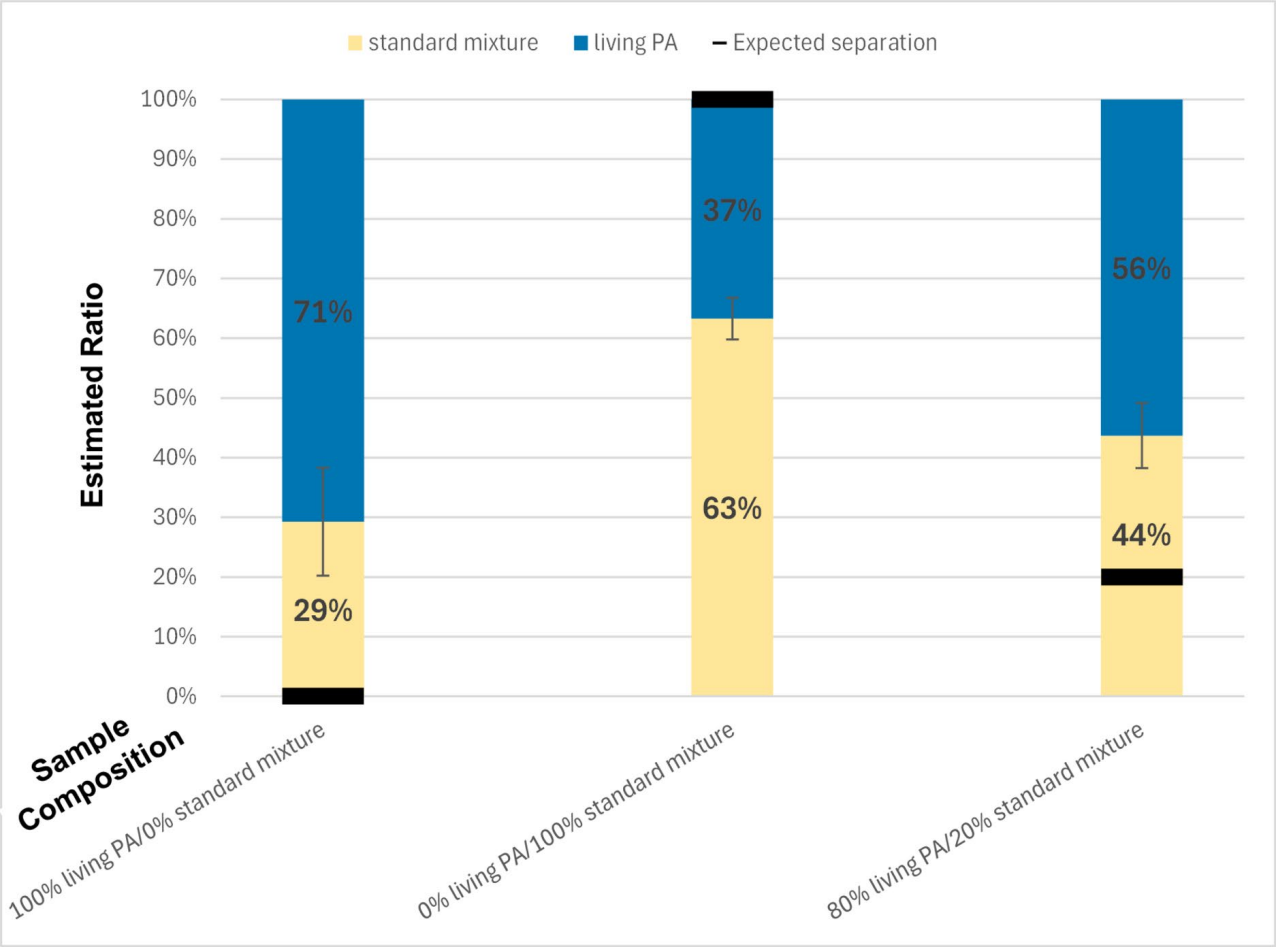
Histogram representation of the results of the classifiers applied on living *P. aeruginosa* and a standard mixture in different ratios. Each bar represents the classification results for a different ratio. The real ratio tested is displayed at the bottom of each column, on the x-axis. Each ratio has been tested on a classifier trained with two classes, living *P. aeruginosa* and the unknown sample called Standard Mixture. Each type of particle (or class) is linked to a specific color. Living *P. aeruginosa* are shown in blue and the Standard Mixture is displayed in light yellow. The black line on each bar indicates the expected separation between the two classes based on the real ratio. The values featured here are the average results obtained over 10 videos per ratio (30 videos in total). The whiskers indicate the standard deviation obtained over the results from the 10 videos of the tested ratio. From left to right, the standard deviations are 9%, 4%, and 5%, respectively. In this graph, PA stands for *P. aeruginosa*.

Notably, the standard mixture may already contain living *P. aeruginosa*, which could contribute to an increased risk of misclassification. However, the first two bars of Fig. 8 indicate that the misclassification rate, ranging from 30 to 40%, is comparable to that in the experiment involving living and dead bacteria, i.e. 30 to 40% of misclassification. Furthermore, a significant shift in the quantity of objects detected as *P. aeruginosa*, from 37% of the *P. aeruginosa* to 56% is observed when comparing the standard mixture and the mixture in which extra *P. aeruginosa* has been added (the right two bars of Fig. 8). These findings provide a proof-of-concept for the validity of our method to detect an increase in the concentration of a specific type of bacteria out of the crowd.

## Discussion

This study aims to assess whether it is possible to differentiate between bacteria only based on their trajectory. We demonstrated that a low-cost optical system coupled with an adapted data processing pipeline enables the discrimination between bacteria, whether living or dead, and beads on the one hand, and, at least for *E. coli* and *P. aeruginosa*, living and dead bacteria on the other hand. As expected, the performance of the detection algorithm is closely tied to the mobility of the studied bacteria: the more active swimming behavior the bacterium tested exhibits, the more accurate the detection is. As a result, *P. aeruginosa* can be easily distinguished from inert objects such as beads. Although more challenging, convincing results are also obtained with *E. coli* and *S. aureus* which exhibit different behaviors compared to *P. aeruginosa*. Although reaching the highest accuracy is important, of course, the key contribution of this paper, as highlighted by the last experiment (Fig. 8), is demonstrating the feasibility of detecting abnormal bacterial concentrations in a sample, despite classification inaccuracy inherent to the method.

The ability of the algorithm to discriminate between dead bacteria and beads proves that, even when dead, bacteria do not behave like inert objects. Comparing misclassification rates and the features’ importance across classifiers reveals that the movement of dead bacteria is slightly weaker than that of living bacteria, but remains sufficient to differentiate them from inert objects. The situation is however different for *P. aeruginosa* and *E. coli* on the one hand and *S. aureus* on the other hand. Even if the misclassification rate is quite high, it is still possible to differentiate living *P. aeruginosa* from a dead *P. aeruginosa*. This suggests that the movement of bacteria changes when they die: it can be assumed that they lose their active movement but keep the passive ones. The conclusion is the same for *E. coli*. However, it is impossible to differentiate a dead *S. aureus* from a living *S. aureus* based solely on the trajectory. As *S. aureus* does not have any active movement when far away from the surface, this observation also makes sense. Several assumptions can be put forward to explain the movement of dead bacteria, such as their shape, their density, or the composition of their membrane. However, further investigations using high-resolution imaging and specific experimental conditions would be necessary to validate or refute this assumption.

As mentioned previously, the error in the mixture preparation is estimated to be 8%, which could explain some discrepancies between the expected values and the results given by the algorithm. However, it cannot explain gaps of 20% and higher observed in some conditions (e.g. dead *E. coli*/beads classifier). The observed misclassification rate can be attributed to several factors, including biological (e.g., intrinsic differences in bacterial behavior or morphology as noted above), physical (e.g., limitations in microscope resolution or loss of focus due to vertical bacterial movement), and computational issues (e.g., improper object or trajectory capture, insufficient data within individual trajectories, false trajectories in the training datasets, or intrinsic errors in the classification algorithm). These sources of distortion highlight several potential avenues for method improvement.

Several avenues for further study and technique improvement have been identified. First, the complete study was conducted at room temperature to match the field application. However, it is well known that for the bacteria tested in this study, their movement is more pronounced at 37 °C^30^. Additionally, experiments have shown that between the preparation of the sample at 37 °C and the end of the image acquisition at room temperature, the sample has time to cool down and bacteria to become less active. Maintaining a stable temperature of 35–37 °C might improve the detection for all bacteria.

The current version of the data processing pipeline relies on very common algorithms for image segmentation, particle tracking, and classification (see Methods for more details). Future work could focus on optimizing these algorithms or replacing them with state-of-the-art alternatives. Segmentation might be improved to reduce its computational time and other deep-learning algorithms such as U-NET^31^ or Cellpose could be tested^32^. Advanced segmentation tools like Cellpose represent promising candidates for the segmentation process. One of the main challenges with Cellpose is the requirement for ground truth annotations to train the model. However, the human-in-the-loop methodology offered by Cellpose greatly mitigates this limitation^33^. Similarly, U-NET detection models and YOLO^34^ often used in real-time applications, are transposable to our context. However, challenges such as detection speed need to be addressed (while segmentation provides high accuracy, it can be computationally intensive and slow, ). Using smaller models and smaller input images (typ. 640 × 480) could improve the speed but this approach requires splitting our high-resolution images (2,464 × 2,056) into smaller sub-images, leading to the risk of losing trajectories that span across multiple sub-images.

The selected particle tracking method does not use any morphological information about the objects to perform. When two particles are close, it can sometimes lead to artifacts in the trajectory. Checking how consistent the tracked particle size or shape is for example, might reduce the number of errors during this process. Testing some of the algorithms provided to the Cell Tracking Challenge^35^ could offer better alternatives or provide ways to improve the algorithm used here. All the alternatives discussed for data processing are avenues for improving detection accuracy. However, it is important to note that they will also impact system response time. These two factors are often at odds, and the trade-off depends heavily on the application. For instance, in applications like infection prevention, achieving a 100% score may not be the priority. Instead, the critical factor is response time: raising an alert in near real-time when a sample deviates significantly from the standard is already a valuable achievement.

The classifier described in this study is quite basic (Random Forest classifier) and relies on eleven features that we consider relevant given the expected behavior of the studied bacteria. Investigation for new relevant parameters might improve the efficiency of the algorithm. Some features commonly used for the detection of diffusion modes^24^ have also been tested. It turns out that they necessitated a longer calculation time, and had a lower efficiency for our application. Integration of other features, e.g. characteristics of random moves, movement of bacteria with respect to the others, or characteristics of the smoothness of the trajectories, can also be investigated. These features may aid in differentiating bacteria like *S. aureus* but may also lead to a significant increase in computation time. Alternatively, testing more complex deep learning algorithms such as Convolutional Neural Networks or Autoencoders could improve the results^24,36^.

In the current version of the processing algorithm, the classification is performed only on trajectory features. Another approach, based on morphological features of the tracked objects has already been demonstrated in a similar context in^16^. In this study, images were acquired with a laboratory microscope. The transposition of that paper’s methods on images acquired with the same homemade microscope as ours had also been tested (data not shown). Results were encouraging but not accurate enough to positively conclude about the relevance of using solely morphological parameters. However, using together morphological parameters and trajectory might be a winning combination to increase the accuracy of the prediction.

Up to now, only three bacteria have been tested. This set of bacteria allowed us to compare three swimming behaviors (very active, active, and passive), two morphologies, two bacteria with similar morphologies but different behaviors, and a bacterium with an inert object of assumed similar behavior. However, other existing pathogens display different behaviors from *E. coli*, *P. aeruginosa*, and *S. aureus*. Furthermore, depending on the application, field samples are likely to contain several species of bacteria at once. The study could therefore be deepened by adding other types of bacteria or bacteria-sized microorganisms, especially the ones that are common in hand microbiom, to bring it closer to actual application cases. It is likely that, given the diversity of objects to identify, the algorithm is no longer able to formally identify one pathogen from another but could still classify pathogens by categories according to the type of their movement, which would already be a very nice breakthrough. Indeed, compared to conventional detection methods outlined in the introduction, our approach offers three main advantages: (i) it eliminates the need for additional consumables, unlike PCR or immunological tests; (ii) it requires only a low-cost microscope and a standard computer for image acquisition and processing, avoiding heavy laboratory equipment; and (iii) it can target a wide range of bacteria, whereas most of the traditional methods are specific to one bacterium or even one specific strain. However, it is important to emphasize that our method is not intended to compete with standard approaches, as the performances and the targeted applications differ significantly.

These results prove that the combination of optical imaging and artificial intelligence is a versatile tool for the detection of potential bacterial contamination. Since the acquisition system is small (40 cm x 20 cm x 10 cm) and low-cost ─ the selling price of a complete device based on this technology being estimated to be €3500 ─ the way is open for the design of embedded systems for the identification of bacterial contamination. Such a system can find applications in a wide range of fields. The set-up used during our experiments already fits in medical devices such as a hand sanitizer dispenser (as suggested in^16^) apart from a computer. However, the computing power required by the algorithm as it exists is reasonable, making a complete integration with a state-of-the-art embedded processing unit possible. As of today, the complete acquisition and analysis pipeline takes about 15 min (on a laptop with an Intel core i7 processor and 32Go of RAM) which is already faster than most other identification techniques. It should be noticed that this processing time is mainly dominated by object and trajectory detection, the time required to calculate the features of the trajectories and the inference time of the models are negligible in comparison.

Finally, the final experiment provides a first proof of concept for the application of this technique to the detection of abnormally high concentrations of a microorganism in any sample. This result could be exploited to create a contamination detection system during hand disinfection comparable to the one described in^16^. Key challenges to transition from the proof-of-concept to a prototype are: (i) the integration of the hardware, even though, as stated here above, the dimensions of the microscope used for this study make this integration possible), (ii) the management of the important quantity of data generated during video acquisition and the computing power required for the processing, (iii) the pre-treatment of the hand-sanitizing sample to extract only particles that can travel through the microfluidic system and can be observed in the microscope’s field of view and (iv) the validation of the whole system in actual conditions.

## Conclusion

This study is a proof-of-concept for identifying bacteria based solely on their trajectories. By focusing on trajectory rather than morphological features, some constraints on the acquisition system are released (e.g. spatial resolution, image quality), which is a requirement to go for a fully integrated detection system. For example, the optical set-up is compact enough to be integrated into a medical device such as a hand sanitizer dispenser. With a suitable data processing pipeline, we highlighted that it is possible to differentiate *P. aeruginosa*, *E. coli*, and *S. aureus*, whether dead or alive, from internal particles. Results also showed that differentiation between living and dead bacteria is also possible for *P. aeruginosa* and *E. coli*. Furthermore, the final experiment provided a proof-of-concept for the detection of an abnormal amount of a given bacteria out of the crowd, which is very promising for the future development of early alert systems preventing the spread of bacterial infections.

## Methods

### Preparation/samples

#### Beads

Spherical latex beads (Invitrogen Nonionic latex beads from ThermoFisher Scientific, reference: N37464^37^) of 1 μm in diameter were used as a simple model for inert objects. Beads were diluted in Phosphate Buffered Saline (PBS) with bacteria to various ratios at a global concentration of 10^5^ beads/mL.

#### Bacterial strains, cultivation and killing

*Escherichia coli* (*E. coli*), K12 strain, was cultivated in Luria Bertani broth, *Pseudomonas aeruginosa* (*P. aeruginosa*), PAO1 strain, was cultivated in Tryptic Soy Broth and *Staphylococcus aureus* (*S. aureus*), clinical strain A170, was cultivated in Tryptic Soy Broth supplemented with 2% (w/v) yeast extract.

For each image acquisition, all bacteria were thawed and grown overnight at 37 °C (stationary phase). For the preparation of dead bacteria, bacteria were heated at 70 °C on a heating block for 30 min before cooling down at room temperature. All bacteria were pelleted and resuspended in PBS. The concentration of each species was estimated with a counting chamber (Petroff-Hausser Counting Chamber from Hausser Scientific, catalog **#** 3900^38^). Samples were created by diluting one or several bacteria or beads in 200 µL PBS with different ratios at a global concentration of 10^5^ bacteria/mL. Treated bacteria were plated with controls and grown overnight at 37 °C to ensure their viability state.

#### Uncertainty in mixture Preparation

The samples prepared for the experiments described all along the paper was made by mixing stock solutions described hereabove. When ratios or percentages are indicated, they have to be interpreted in terms of number of bacteria per milliliter, not in volume. Due to uncertainty in the particle counting in stock solution as well as pipetting errors when diluting the stock solution and when mixing them, a non-negligible uncertainty exists between the targeted ratio and the actual ratio. This uncertainty can be estimated as follows.

Using error propagation rules, we can estimate that when diluting $\:a$ fractions of the stock solution in $\:b$ fractions of the solvent, the new concentration of solution is $\:a/(a+b)$ with an uncertainty $\:{u}_{dill}$ given by:

$$\:{{u}_{dill}}^{2}=\frac{{{u}_{b}}^{2}}{{a}^{2}}+\frac{{b}^{2}{{u}_{a}}^{2}}{{a}^{4}}$$

where $\:{u}_{b}$ and $\:{u}_{a}$ are the pipetting error estimated at 5%. The second source of uncertainty is the counting error $\:{u}_{count}$, which was estimated at 1% with the counting chamber we used. Combining dilution and counting error leads to an estimation of the uncertainty in the particle concentration for each solution to mix given by $\:u=\sqrt{{{u}_{dill}}^{2}+{{u}_{count}}^{2}}$. Finally, the uncertainty on mixture ratios was estimated using the worst-case method, i.e. by considering that the error in the concentration of particles in one compound is maximal whereas it is minimal for the second.

Putting everything together leads to an estimation of the ratio of about 8% for all mixtures composed of 30% of one type of bacteria or bead and 70% of another type of bacteria or beads.

### Hardware

#### Optical set-up

Recordings were made with a homemade microscope combining a ZEISS N-Achroplan 40x/0,65 M27 objective^39^ and a Ximea MC050MG-SY camera^40^. Videos were acquired with the Ximea software provided with the camera. Videos of around 30 s with a maximal frame rate of 8 frames per second were recorded, each frame having an image resolution of 2,464 × 2,056 pixels and a corresponding field of view of 340 × 283 μm², leading to a resolution for the microscope of 135 nm/pixel.

#### Microfluidic chips

Samples were imaged through a microfluidic chip (µ-Slide I Luer 0.2 mm uncoated from Ibidi, catalog number: 80171^41^). Samples were pipetted in a chip to fill it as much as possible, then plugs were used to seal the chip. The sealing has two main consequences: (i) it avoids contamination of hand by the sample, and (ii) as the chip is filled, it avoids rapid movements of the sample in the channel. In addition, no external source inducing movements or flow such as pumps were used during image acquisition to avoid parasitic movement of the pathogens.

### Computer

All algorithms were executed on a Dell Latitude 5410 laptop with a 4-core i7-106110U processor clocked at 1.8 GHz with 32GB 2666 MHz DDR4 RAM, a 512GB SSD hard drive, and no graphics processor other than that on the motherboard.

### Algorithm

#### Segmentation

Acquired videos were transformed using a self-made Python script and functions from the scikit-image 0.19.3 library^42^. Each frame was segmented individually. First, a Gaussian filter^43^ was applied to remove noise (Fig. 2B). Then, a Sobel filter^44^ was applied to detect the edges of the objects in the picture. After simple thresholding, the obtained binary image (Fig. 2C) was used with a watershed algorithm^45^ to detect the different particles in the pictures (Fig. 2D). For each object detected in every frame, the x and y-coordinates were registered in a file with the name and the number of the frame. The coordinates from all the objects of one video were compiled in one unique file.

It was common that undesirable particles (dust, fiber, beads, bacteria) to appear on images. These particles usually appeared on every frame and were not moving at all. These particles had to be removed for at least one of two reasons: (i) their trajectories were not representative of the swimming behavior of particles we were imaging, and (ii) they were not particles we wanted to image. Thus, for each video, 10 frames were randomly chosen to create an average image. As the non-moving particles were on most of the frames, they appeared on this average image whereas moving particles did not. This average background was segmented, and particles detected at the same position in the binary mask for each frame were removed.

#### Particle tracking

Once the segmentation is done, the resulting file is processed by an existing tracking algorithm (ImageJ particle tracker 2D/3D)^46,47^ to build back the objects’ trajectories. Objects could disappear in one frame and reappear in the next. To overcome this issue, the algorithm looked for disappearing objects over the 3 next frames. The algorithm was set to build Brownian trajectories (instead of rectilinear ones) of an average movement of 20 pixels between each frame. Each trajectory was given a number. The frame number, and the x- and y-coordinates of the object were registered in a table for every trajectory. All the trajectories of one video were computed in a single file. The results of the tracker are presented in Fig. 2E for *E. coli*. Each colored line corresponds to one trajectory.

The trajectories created could be as short as two points (an object detected over two frames and never detected again). Trajectories with a low number of points do not contain enough information to be taken into account. Moreover, the more points in a trajectory, the more representative of the global behavior of the bacteria the trajectory. Thus, a threshold of 50 points per trajectory has been implemented and trajectories under this threshold were removed from the dataset.

Even though no flow was induced during the measurements, a residual flow from the manipulation could still exist. This privileged direction could be used as a discriminant factor for the algorithm or hide the objects’ movements. To counteract this, the average object’s displacement between 2 successive frames is calculated by averaging the movement calculated for all objects detected in the image. The resulting displacement is then subtracted from the migration calculated for each object.

#### Dataset Preparation

For each experiment and each sample, 10 videos of around 30 s were acquired. To obtain sufficient relevance, 3 biological replicates were made for each experiment. Thus, 30 videos per sample were gathered. During the different experiments, three learning and verification datasets were required for each bacterium, one between living bacteria and beads, one comparing dead bacteria and beads, and one of living bacteria against dead bacteria of the same species. The size of the datasets has been adjusted to have a balanced number of trajectories for each type of object for the training, and enough (at least 30% of the total amount of trajectories) remaining trajectory for the validation.

For *P. aeruginosa*, the living/beads, dead/beads, and living/dead classifiers have been trained with 456, 1,000, and 825 trajectories per class respectively. The corresponding verification datasets were composed of 226, 494, and 407 trajectories per class respectively. The living/beads classifier was then tested on 9,685 trajectories (1,417 for *P. aeruginosa*, 4,268 for beads, 2,273 for the 70/30 ratio, and 1,727 for the 30/70 ratio). The dead/beads classifier was tested on 17,653 trajectories (2,983 for *P. aeruginosa*, 5,512 for beads, 4,116 for the 70/30 ratio, and 5,042 for the 30/70 ratio). Finally, the living/dead classifier was tested on 11,535 trajectories (2,455 for living *P. aeruginosa*, 2,993 for dead *P. aeruginosa*, 2,699 for the 70/30 ratio, and 3,388 for the 30/70 ratio).

Regarding *E. coli*, the living/beads, dead/beads, and living/dead classifiers have been trained with 456, 1,664, and 1,187 trajectories per class respectively. The corresponding verification datasets were composed of 226, 820, and 585 trajectories per class respectively. The living/beads classifier was then tested on 13,132 trajectories (2,376 for *E. coli*, 4,268 for beads, 3,678 for the 70/30 ratio, and 2,810 for the 30/70 ratio). The dead/beads classifier was tested on 47,003 trajectories (4,943 for *E. coli*, 19,636 for beads, 7,753 for the 70/30 ratio, and 14,671 for the 30/70 ratio). Finally, the living/dead classifier was tested on 29,271 trajectories (10,843 for living *E. coli*, 4,075 for dead *E. coli*, 8,333 for the 70/30 ratio, and 6,020 for the 30/70 ratio).

Regarding *S. aureus*, the living/beads, dead/beads, and living/dead classifiers have been trained with 456, 1,878, and 6,204 trajectories per class respectively. The corresponding verification datasets were composed of 226, 925, and 3,056 trajectories per class respectively. The living/beads classifier was then tested on 12,063 trajectories (3,095 for *S. aureus*, 4,268 for beads, 2,009 for the 70/30 ratio, and 2,691 for the 30/70 ratio). The dead/beads classifier was tested on 69,283 trajectories (19,355 for *S. aureus*, 19,636 for beads, 17,058 for the 70/30 ratio, and 13,234 for the 30/70 ratio). Finally, the living/dead classifier was tested on 119,922 trajectories (34,257 for living *S. aureus*, 24,994 for dead *S. aureus*, 32,913 for the 30/70 ratio, and 27,758 for the 30/70 ratio).

For the identification experiment, an extra learning dataset containing 961 trajectories per class between living *P. aeruginosa* and the standard mixture was created alongside a verification dataset of 474 trajectories per class. This classifier was tested over a dataset containing 1454 trajectories for *P. aeruginosa*, 2990 for the standard mixture, and 1910 trajectories detected in the sample containing the mixture composed of 80% of *P. aeruginosa* and 20% of the standard mixture.

All classifiers were tested over the most data possible for each sample including data contained in the learning and verification datasets.

#### Classifier

For the classification task, the objects tested are the trajectories, the input features are some metrics described in more detail below that characterize them, and the classes are the type of objects to distinguish (living bacteria, dead bacteria, beads). Objects change from one experiment to the other but features are the same for all of them. Classes depend from one experiment to the other. The classifier used here was a Random Forest Classifier^48^. The scikit-learn 1.1.1 Python module has been used to implement this classifier^49^. Three hyperparameters of the algorithm were adjusted to optimize the learning and classification process: the number of decision trees in the forest (*n_estimators*), the maximal depth of the tree (*max_depth*), and the maximum number of leaf nodes a decision tree can have (*max_leaf_nodes*). The parameters were set at 100, 25 and 30, respectively for all trained classifiers. These parameters have been set to decrease the misclassification rate while avoiding overfitting.

The eleven features used for the classification are:

1. The trajectory length, which is the number of frames on which the trajectory is visible.
2. The average speed, which is the average distance in pixels between the position of the object in two consecutive frames. As its name suggests, average speed characterizes how fast the object moves;
3. The standard deviation of the speed, which is the standard deviation of the distance in pixels between the position of the object in two consecutive frames. This feature describes how uniformly the object moves all along the trajectory;
4. The speed slope, which is the steering coefficient of linear regression of the distance in pixel between the position of the object in two consecutive frames with respect to the time. This feature can be used to describe if an object tends to accelerate or decelerate during its movement.
5. The total angle, which is the sum of the angles calculated between two consecutive points of the trajectory. It describes the tendency for the object to make circular trajectories like circles or helices, etc….
6. The angle standard deviation, which is the standard deviation of the angle calculated between two consecutive points of the trajectory. This feature describes how uniform the potential rotational movement occurs.
7. The total angle slope, which is the steering coefficient of regression of the angle calculated between two consecutive frames with respect to the time. This feature indicates whether the circular movement is uniform or whether it tends to accelerate or decelerate along the trajectory.
8. The error towards linear function, which is the sum of the distance between the actual points of the trajectory and a linear trajectory between the first and last point of the trajectory. This feature indicates how the movement of the particle is close to the straight line.
9. The square error towards linear function, which is calculated in the same way with square errors is instead of absolute errors. Compared to the previous feature, this one gives more weight to large gaps between the actual trajectory and the straight line.
10. Semi-major axis, which is the semi-major axis of the elliptic regression of the trajectory. This feature indicates to what extent a trajectory can be compared to an ellipse and if so, what is its semi-major axis. For non-elliptic trajectory, this feature tends to infinity.
11. Semi-minor axis, which is the same as the semi-major axis.

After a random forest has been trained, scikit-learn can measure the importance of each feature. This metric is defined as the frequency of use of a given feature in all the decisions made by all trees of a Random Forest. Feature importance is expressed in %. The higher this value, the more the feature can be considered as discriminating.

## Data Availability

The datasets generated and/or analysed during the current study are available in the Open Science Framework repository - https://osf.io/s8k3v/.
